# Redefining value: a discourse analysis on value-based health care

**DOI:** 10.1186/s12913-020-05614-7

**Published:** 2020-09-14

**Authors:** Gijs Steinmann, Hester van de Bovenkamp, Antoinette de Bont, Diana Delnoij

**Affiliations:** Erasmus School of Health Policy and Management, Erasmus University Rotterdam, PO Box 1738, Rotterdam, 3000 DR The Netherlands

**Keywords:** Value-based health care, Discourse analysis, Shared decision-making, Ambiguity, Netherlands

## Abstract

**Background:**

Today’s remarkable popularity of value-based health care (VBHC) is accompanied by considerable ambiguity concerning the very meaning of the concept. This is evident within academic publications, and mirrored in fragmented and diversified implementation efforts, both within and across countries.

**Method:**

This article builds on discourse analysis in order to map the ambiguity surrounding VBHC. We conducted a document analysis of publicly accessible, official publications (*n* = 22) by actors and organizations that monitor and influence the quality of care in the Netherlands. Additionally, between March and July 2019, we conducted a series of semi-structured interviews (*n* = 23) with national stakeholders.

**Results:**

Our research revealed four discourses, each with their own perception regarding the main purpose of VBHC. Firstly, we identified a Patient Empowerment discourse in which VBHC is a framework for strengthening the position of patients regarding their medical decisions. Secondly, in the Governance discourse, VBHC is a toolkit to incentivize providers. Thirdly, within the Professionalism discourse, VBHC is a methodology for healthcare delivery. Fourthly, in the Critique discourse, VBHC is rebuked as a dogma of manufacturability. We also show, however, that these diverging lines of reasoning find common ground: they perceive shared decision-making to be a key component of VBHC. Strikingly, this common perception contrasts with the pioneering literature on VBHC.

**Conclusions:**

The four discourses will profoundly shape the diverse manners in which VBHC moves from an abstract concept to the practical provision and administration of health care. Moreover, our study reveals that VBHC’s conceptual ambiguity largely arises from differing and often deeply rooted presuppositions, which underlie these discourses, and which frame different perceptions on value in health care. The meaning of VBHC – including its perceived implications for action – thus depends greatly on the frame of reference an actor or organization brings to bear as they aim for more value for patients. Recognizing this is a vital concern when studying, implementing and evaluating VBHC.

## Background

Today’s remarkable popularity of value-based health care (VBHC) is accompanied by considerable ambiguity concerning the very meaning of the concept. Several scholars have noted this ambiguity, with explanations ranging from the concept being diluted in academic literature [1], to VBHC being a highly ambiguous concept in and of itself [2], and to VBHC being adopted and adapted within various local contexts [2, 3]. This paper elicits an alternative conception. We aim to map the ambiguity by examining how VBHC is discursively framed and explore the presuppositions that give shape to diverging rationales. Our qualitative study fills a literature gap by conducting a discourse analysis specifically focused on the various ways VBHC is interpreted, thereby also contributing to a better understanding of VBHC in general, and some of the challenges that lie ahead regarding implementation efforts.

### The origins of VBHC

It feels safe to say that the core principles of VBHC are laid out in *Redefining Health Care* (2006) by Michael Porter and Elizabeth Teisberg [4]. They argue that value in health care consists of what matters most to patients: the health status they achieve (outcomes) and the price they must pay for it (costs). Therefore, providers should focus on generating maximum value for their patients by helping them achieve the best possible outcomes and by doing so in a cost-efficient way. Importantly, value is created at the level of medical conditions, over full care cycles [4(p99–105)]. Providers should structure their organizations in alignment with the goal of value: forming integrated practice units whose dedicated work focusses on one or a few related medical conditions, or specific patient groups, with coordination over the full cycle of care [4(p167–77)]. Payment structures should also be aligned with value: bundled payments should cover full cycles (or episodes) of care [4(p265–67)]. Perhaps most importantly (according to Porter and Teisberg), providers should start measuring and reporting outcome data on each of the medical conditions they treat [4(p7)]. The widespread availability of outcome information will enable professionals to learn, to improve, and to refer patients to the providers that perform best. Moreover, this will unleash the right kind of competition, the kind that is based on value. When providers compete on value they will have to demonstrate good health outcomes at a competitive price in order to attract patients, which means they are also compelled to work as efficiently as possible [4]. The general idea is that ‘if value improves, patients, payers, providers, and suppliers can all benefit while the economic sustainability of the health care system increases’ [5].

### The ambiguity surrounding VBHC

The conceptual ambiguity surrounding VBHC is conspicuously evident in academic publications. To some scholars, VBHC is primarily a ‘management concept’ [1] or a ‘management innovation’ [2, 6]; to others, it is basically a business ‘strategy’ for both providers and payers [7]; others see it as a ‘governance regime’ [3], or a ‘health policy framework to integrated care’ [8]. Additionally, while the importance of outcome measurements for VBHC is generally well established, the range of its utility remains debated. Whereas some argue that outcome measurements seem less valuable regarding chronic diseases [9], others argue that such measurements are actually particularly applicable to chronic conditions [10]. Similar dissonance can be observed regarding the idea of value-based payment: while some scholars include pay-for-performance and capitation as value-based methods [11], these payment models are explicitly declared invalid by others [4]. Against this background, some suspect VBHC to be another one of those management concepts in health care (e.g. like Lean), whose promising start eventually grows into little more than a buzzword [1].

Perhaps it should not come as a surprise that underneath this cloak of ambiguity, VBHC has been adopted and implemented in fragmented and multifarious ways [2, 9, 12]. These developments may well contribute to the ambiguity. In fact, the observation that the implementation of VBHC requires the concept to be ‘translated’ (i.e. adopted and adapted) into multiple local contexts is indeed brought forth to explain the ambiguity [2, 3]. Another, perhaps slightly provocative explanation, is that the meaning of VBHC is being diluted due to a lack of understanding by knowledge producers, particularly within academic writings [1]. Alternatively, it has also been stated that VBHC is a highly ambiguous concept in and of itself [2].

Although the above-mentioned accounts may well be part of the story, we claim that a more profound comprehension can be found. We argue that all concepts, including VBHC, acquire meaning within a frame of reference [13–15]. An important part of the ambiguity that surrounds VBHC is that the concept is being perceived differently within different frames of reference. These frameworks of perception are often founded upon deeply rooted presuppositions and convictions. An important aim of this study is to explore the underlying assumptions that give shape to various interpretations of VBHC.

We thus investigate and map the ambiguity that surrounds VBHC by conducting a discourse analysis on VBHC in the Netherlands. Our main question is: *How is VBHC being interpreted by actors and organizations that monitor and influence the quality of care in the Netherlands*?

The Netherlands forms an interesting setting, as its healthcare system is based on regulated competition, and the concept of VBHC is currently being adopted by a variety of organizations, including national policy institutions. Moreover, outcome measurements form an important theme within Dutch health policy. There is, however, an ongoing debate between various stakeholders on the use and public disclosure of these outcome data. Therefore, it is both relevant and timely to explore the interpretation of VBHC in the Netherlands.

### The Dutch context

In 2006, the same year in which Porter and Teisberg published *Redefining Health Care*, the Dutch Health Insurance Act came into force. This was a significant regulatory overhaul. By law, private insurance companies were now charged with the task of stimulating the quality and efficiency of healthcare providers (mainly through selective contracting); while competition for members among insurers should refrain them from excessively increasing annual premiums. This entailed an increased demand for adequate quality information, which would allow all participants in the system (including patients and government agencies) to usefully compare and evaluate providers (and insurers). Since 2014, healthcare providers are legally required to report quality information to the National Healthcare Institute. In recent years, outcome measurements are increasingly becoming part of this requirement.

## Methods

We have conducted a discourse analysis that aims to map the various ways in which VBHC is being interpreted in the Netherlands. Discourse analysis is a particularly well-suited approach to uncover the foundational presuppositions that shape the rhetorical use of ambiguous concepts [16, 17].

Within this study, ‘discourse’ refers to: a set of statements and expressions regarding a certain issue (e.g. VBHC); conjoined by shared assumptions (which are not always expressed explicitly); often framing the way people think, talk and write about that issue; with the potential to guide actions and decisions [17, 18].

Although such an understanding recognizes that discourses may frame action, we do *not* depart from a deterministic conceptualization of discourse [19]. Additionally, this paper does *not* elaborate on the power/knowledge relations that may harness discourses and dictate social realities – as is customary in (Foucauldian) critical discourse analysis [20, 21]. Rather, this study departs from the presupposition that all concepts, including VBHC, acquire meaning within a particular frame of reference [13, 14]. We examine how certain (deeply rooted) assumptions and frames of reference generate particular interpretations of VBHC.

We have conducted our discourse analysis accordingly: analyzing statements and expressions regarding VBHC, which were gathered through semi-structured interviews (*n* = 23) and document analysis (*n* = 22). Thereby building on the notion that discourses materialize in such textual representations [17, 22, 23].

### Selection

Our study attains a *national* orientation: we are interested in national discourses. This focus on national debates is particularly relevant regarding the Dutch healthcare system: the country has a centralized health policy framework, in which several influential sector associations, which represent particular stakeholders, operate on a national level. Therefore, through purposive sampling [24], we selected our documents and interviewees with the aim to gather statements and expressions that are relevant on a national scale.

Between March and July 2019, we have conducted 23 semi-structured interviews with representatives of government bodies (the Ministry of Health, Welfare and Sports, and the National Health Care Institute), national branch/sector associations, several representatives of insurer companies, and provider representatives (mostly members of the executive or supervisory board). In addition, two of the interviewees are academics specialized in health policy and management in the Netherlands. The interviews were audio recorded and lasted 55 min on average, with verbatim transcripts averaging 7000 words. See Supplementary file 2 for our interview guide (developed for this study).

As mentioned, we complemented our interviews with document analysis, thereby diversifying our dataset, which should strengthen our findings. Our national orientation also shaped the document selection: we searched for *official publications*, that are publicly accessible, established by nationally active actors and organizations. The respective documents (*n* = 22) were all searched for online, selected based on their relevance to the topic (VBHC), and their national orientation. Only recently published documents were considered applicable, in order to avoid as much as possible, the possibility that the statements in the documents were related to structurally different circumstances and events than the interviews. Resultantly, the ‘oldest’ document consists of a ministerial letter to parliament from October 2015; a text we considered too relevant to exclude. We halted our search for additional documents in December 2019, when our data-analysis reached a point of saturation regarding our main question.

### Analysis

QDA software (Atlas.ti) was used for structuring the analysis. Roughly speaking, the process of analysis consisted of three stages. Firstly, we developed an initial coding scheme [24], which was established through the open thematic coding of the first five interviews. The main goal of this scheme was to develop a structural set of codes – with brief descriptions of the cluster of themes and topics that fall under each code – which would be utilized to label the data, enabling a structured and focused analysis with deliberate comparison in and between texts. The coding scheme was developed by GS, in correspondence with the feedback received from HB, DD and AB.

The second stage was initiated by the increased amount of data and characterized by the sequential re-reading of the texts and the re-evaluation and adaption of the coding scheme. Transcripts and documents were co-read by HB.

In the third stage, the process of coding increasingly became intertwined with interpretation. This fully launched the analysis of the coded texts: guided by the main research question – and informed by our definition of discourse – we searched for patterned expressions and conjoined lines of reasoning, including a specific search for coherence and discordance. Particularly during this stage, the main author wrote regular analytical reports which were reviewed by each author and discussed in team sessions. See Supplementary file 1 for additional insight into our methods.

## Results

Our analysis revealed four diverging discourses on VBHC in the Netherlands. Interestingly, shared decision-making (SDM) is a core part of VBHC, in all four discourses. Before elaborating on this second observation, let us first describe the different discourses (see Table 1 for an overview).

**Table 1:** Four discourses on VBHC

	PEMP	GOV	PROF	CRI
**VBHC**	Framework for improving patients’ position regarding medical choices	Toolkit to steer and incentivize providers	Methodology for optimizing healthcare delivery	Dogma of manufacturability
**Assumption**	Patients in disadvantaged position, inequality in patient doctor relation	Incentives can improve behavior of medical professionals	Professionals intrinsically motivated to serve patients interest and deliver value	Health care is too complex for standardized value
**Main use of outcome info**	Choice information (for patients)	To stimulate professionals	Professional learning and improving	Learning and within patient-doctor relation
**SDM**	End goal: would require and demonstrate empowered patients.	Way to enable patients to act upon outcome information.	Way to improve care delivery and create value for individual patient.	Great: addressing individual needs in cooperative relation.

### Four discourses on VBHC

Firstly, there is what we have labeled a *Patient Empowerment* discourse (PEMP), in which VBHC is chiefly portrayed as a framework for strengthening the position of patients regarding their medical decisions. Secondly, we have identified a *Governance* discourse (GOV) in which VBHC is primarily construed as a mechanism to steer and regulate care providers toward value for patients. Third, there is a *Professionalism* discourse (PROF), in which VBHC is predominantly construed as a methodology for the organization and improvement of health care delivery. Fourthly, we have identified a *Critique* discourse (CRI), which is characterized by a specific form of critique of VBHC, particularly its emphasis on measurement and standardization.

#### Patient empowerment (PEMP)

Within PEMP, the interests of patients are cardinal, and a central premise is that VBHC can be an important framework in addressing these interests:

Look, if you believe in adding *value*, then that means that you *optimally* include the patient in the course of events. That you optimally involve them in the story. That is the basis. So, and that is quite difficult, is the patient being taken seriously enough? I see a lot of places in health care where that simply doesn’t happen enough (IP 7).

Moreover, this discourse builds on the viewpoint that the position of patients within the Dutch healthcare system requires improvement. The following quote by former minister Schippers nicely illustrates this notion:

In the past, the position of patients regarding healthcare insurers and doctors was weak. […] Information about the quality of the care that was delivered was also unavailable to these patients. [T]his requires enormous catching-up [25].

In this line of reasoning, patients can and should be empowered by providing them relevant information that can help them in their medical decisions, e.g. which provider and what treatment. Accordingly, PEMP frames information provision as a moral obligation, something patients are entitled to:

I see that as my right. Yes, and based on that [information] I am supported to make a good choice for one provider versus another one who is just not performing as well. [I]f there is data available that shows that one is better than the other, shouldn’t I simply be able to choose that one? (IP 17).

Within PEMP, the capacity to *choose* becomes a goal in itself. Choice empowers patients and should be facilitated through adequate access to relevant information. Therefore, this discourse advocates an increased provision of relevant information to patients:

The starting point for this next phase is that more information becomes available […] so that the patient can better choose ‘which consultation room he or she will end up in’ and that ‘in that consultation room the right decisions can be taken jointly’ [26].

Enabling patients to choose for themselves is thus seen as a step forward; an improvement from past times when ‘patients took the doctor’s advice for granted’ [25]. A presupposition underlying this discourse is that the advice given by doctors will not always match the interests of patients. Therefore, the position of patients requires strengthening, and VBHC is discursively portrayed as a strategy toward patient empowerment.

#### Governance (GOV)

Within the governance discourse (GOV), VBHC is primarily adopted as a mechanism to steer and incentivize care providers toward better outcomes and lower costs (i.e. higher value). A core premise is that the incentives that are built into healthcare systems influence provider behavior. This entails the notion that external parties such as government agencies and insurance companies can and should incentivize providers. Relative to the other discourses, within GOV the financial side of health care gains significance, also regarding VBHC:

The more you produce, the more money you get paid. That is an incentive that does not necessarily lead to more value for the patient (IP 5).

Within GOV, steering efforts to induce improvements are deemed desirable. Particularly financial incentives and payment structures such as bundled payments are seen as a useful tool in generating optimal care delivery. This idea of incentivizing applies to individual providers but also concerns more structural phenomena regarding the organization of health care.

With bundled payments, you will also see that you get different organizational relations. Now it [money] goes either to the gynecologists or to the midwives, and with such a bundled payment it goes to the gynecologists and the midwives, and you have to arrange that as efficiently as possible. [T]hen you’ll get another conversation. Yes, I certainly believe that. So, I think (I thought about it this morning) yes health care is still very strongly supply-driven. That is still the case. We pretend it isn't, but it is. Particularly in maternity care I can see this: we actually know that we should organize it differently, but we just don't do it. Because of our own work enjoyment; our own wallet; and so on (IP 4).

For GOV, VBHC seems like a particularly well-suited framework to incentivize and steer care providers, since the idea of value *combines* outcomes and costs. In doing so, VBHC simultaneously addresses cost-efficiency and quality of care, and thus merges two orientations that are sometimes thought to be at odds with one another. Within this line of reasoning, VBHC establishes a ‘common language’ (IP 20) for external parties and the providers they regulate.

So VBHC is a new bridge, to let economists and clinicians talk to each other. That is part of what VBHC is (IP 25).

The *Governance* discourse presupposes that medical professionals will not automatically work toward optimal value. This assumption is comparable to the previously mentioned presupposition in PEMP (that the interests of medical professionals will not automatically match those of patients), however, in GOV the answer is not empowering patients, but incentivizing professionals. In this light, VBHC becomes a discursive framework to align incentives with value for patients.

#### Professionalism (PROF)

While within PROF it is recognized that the perspective of patients should indeed be more adequately addressed, this notion is nested in a broader understanding of VBHC as a model for organizing and improving healthcare delivery. With the objective of patient value in mind, VBHC constitutes a methodology in healthcare facilities. Crucial to this value-based framework is an emphasis on continuous improvement.

[W]hat is of paramount importance to me about actually implementing the VBHC methodology, is that you create a culture in which our medical professionals are constantly searching to improve the value for their patient, in a data-driven way. […] A culture where you’re constantly looking for improvement (IP 5).

An important element of VBHC as a methodology and a culture of continuous improvement is a renewed focus on medical conditions as the relevant organizational units (as opposed to the traditional medical specialties).

Organizing care around the condition instead of the specialty. In order to provide value to patients, health care needs to be organized differently […]. By multidisciplinary teams, around conditions, through the entire chain and from the perspective of the patient [27].

It is noteworthy that PROF generally stands highly enthusiastic toward VBHC, yet within this discourse, some level of restraint is also advised. Essentially, it is argued that for VBHC to reach its full potential, we must be careful not to let it become an ‘economic’ or ‘management’ tool [27], but instead preserve the alignment with the *intrinsic motivations* of professionals – to improve the quality of care.

If we’re going to impose this from the outside, top-down, again through some nationwide program […] then we won’t make it (IP 15).

In general, PROF considers the intrinsic motivations of medical professionals to be aligned with the interests of patients: to deliver the best possible care. The idea that we need external parties to incentive professionals is therefore refuted:

That’s the idea of VBHC, that you still need to give professionals an incentive to do what’s good – everyone wants to do good (IP 16).

The Federation of Medical Specialists (2017) further illustrates this conviction when they portray their vision for the future:

In 2025, all parties involved in care and well-being will work together in a healthcare system in which the needs of the patient serve as the starting point. For most healthcare professionals, this goes without saying, and is already the starting point for the work they do. Unfortunately, many feel that the healthcare system with its rules and protocols prevents them from providing optimum healthcare and being able to meet the needs of the patient [28].

So, whereas the previous discourses (PEMP and GOV) presuppose that it will require additional efforts to secure the alignment of the choices and motivations of medical professionals with the interests of patients and the goal of value, the underlying assumption of PROF is quite the opposite: it is external regulations that hamper the optimal utilization of professionals’ intrinsic motivations. From this viewpoint, optimizing patient value requires the facilitation of professional expertise through interprofessional learning and improvement. It is within this line of reasoning, that VBHC is discursively framed as a methodology for healthcare delivery.

#### Critique (CRI)

Whereas the previous three discourses all portray a positive attitude toward VBHC, the fourth discourse we identified takes a highly critical stance. This critical discourse (CRI) goes beyond being worrisome about some of the potential disadvantages of VBHC (as we have seen within PROF); it rebukes some of its core principles. Within CRI, VBHC is portrayed as a dogma of manufacturability, which falls short in recognizing the immense intricacies in health care. In particular, the critique concerns the emphasis that is placed on aggregated measurements:

Look, if you ask me ‘what do you see as the biggest disadvantage of that VBHC concept?’ – that is that it *thinks* that value can be measured at the group level. And also, that it is really based on the idea that you can grasp *the good* – good care – in a couple of numbers. That’s a second (IP 16).

CRI not only rebukes the emphasis VBHC puts on standardized outcome measurements; their critique goes deeper: they question the validity of largescale standardization in health care by linking it to a belief in the manufacturability of good care. This belief, that the highest quality care can be measured and (re)produced systematically, is mistaken according to CRI:

[T]hat *focus* on those outcomes, that is a type of utopia of manufacturability. An […] approach like ‘something is only good when its consequence is *measurably good*.’ Well, I think that is a reduction of reality. […] The good is much richer than merely numbers (IP 16).

Instead of assuming that with aggregated data medical professionals can manufacture value, CRI advocates a more personal approach that recognizes the importance of relationships:

It really all departs from the same presumption. Namely: if we have the right instrument, then we will change reality. And that is what I myself always object to. Against those means-end reasonings. As if the context, the preferences of people, are casually put between brackets. They don’t count for the moment. While I think that if there is anything in health care, then it is that the care is made in the relation itself. The relation between patients and practitioners, or between practitioners themselves (IP 22).

Within this line of reasoning, instead of aiming for standardization and comparability, healthcare delivery should attend to differences. The medical ‘attention that people need can be different for each person’ (IP 16). Health care should thus be personalized. However, ‘that personal focus of care is lost in the measurement at group level’ (IP 16).

I think there should be much more of that individualization: what does it mean for me? And that’s not the same as absorbing my preferences into an aggregated dataset, and then saying, ‘oh you belong to that group, and with that group, we will do this’ (IP 22).

Within CRI some of the core premises of VBHC are refuted. Particularly the emphasis on aggregated and standardized measurements is seen as undesirable to health care delivery. A better approach would be to recognize relational complexities and to further personalize care. CRI thus presupposes that health care – and the people that organize, deliver, and receive it – forms a sphere of intricacies that cannot be adequately addressed by an approach that departs from a standardized notion of value. It is from this presupposition, that VBHC is discursively framed as a dogma of manufacturability.

### Divergencies on outcome measurements

While each discourse recognizes the important role of outcome measurements regarding VBHC, this issue – what to do with outcome information – nevertheless remains highly contested. Indeed, it is particularly in relation to outcome measurements that the discourses exemplify conflicting lines of reasoning.

#### Patient empowerment on outcome information

Within PEMP, the purpose of outcome information is first and foremost to strengthen the position of patients in relation to medical professionals and the workings of the healthcare system. Highly illustrative for this line of reasoning is the title of a 2018 report by the National Healthcare Institute: ‘*More control for patients through more outcome information*’ [29].

As mentioned, within PEMP, it’s the ability to make an informed choice that empowers patients – outcome data is seen as particularly useful in this regard:

If good outcome information is available, the patient and caregiver can together make a choice regarding the diagnostics and treatment that is most suitable for that patient. Outcome information is also needed to choose a care provider that meets the wishes of the patient [29].

As you may recall, PEMP states that it is a patient’s right to have access to relevant information. Therefore, such information must become widely available and, furthering this logic, PEMP advocates transparency of outcome measurements:

Transparent, meaning public, outcome information helps patients in choosing a healthcare provider, choosing a treatment together with the practitioner, and […] must, therefore, be available […] to provider *and* patient [29].

To recap PEMP’s view, the position of patients should be strengthened through more adequate information provision, which will encourage better choices. Outcome information, publicly accessible, is framed to be a means to this end.

#### Governance (GOV) on outcome information

Within GOV, outcome information is portrayed as an important tool to incentivize providers. The following quote, from a publication of an insurer company in which they outline their vision, is highly illustrative:

We stimulate higher quality, with better outcomes of care as the central focus. […] Based on our value-oriented approach, we have, for a few years, increasingly been making tangible agreements on (among others) quality measurement, quality comparison and quality improvement in relation to cost. This requires clear outcome indicators [30].

Although the idea of transparency of outcome data is generally seen as positive within GOV, it is stated that making outcome information publicly accessible should serve a function. Keeping in mind the aim to stimulate improvements, GOV questions whether largescale transparency of outcome measurements will be the best way forward. Existing quality registries (which in the Netherlands are *not* open to the public) may already have the desired, incentivizing effect.

This is already going for years, of course, because we’ve had quality registries for years. […] And yes indeed, then you want to do it just as good as the neighbor. So, it is more like an incentive, indeed, to do it better. But we’ve been seeing this for years: that is the effect of those quality registries (IP 2).

In sum, GOV frames outcome information as an instrument to incentivize professionals. Transparency can be desirable as long as it is functional. Nevertheless, even without transparency, GOV construes outcome information mainly as a tool to stimulate providers.

#### Professionalism (PROF) on outcome information

Whereas some advocate (mandatory) transparency of outcome information, PROF displays a different mode of reasoning. Although it is recognized that patients should indeed be optimally informed, it is predominantly *within the doctor’s office* that this optimization should take place.

The efforts should primarily be aimed at making useful outcome information available in the doctor’s office and the conversation between patient and doctor [27].

Within PROF though, the main purpose of outcome data is to enable the continuous improvement among medical practitioners, its use within the doctor’s office is the next step.

We first started to use it, and this still has the emphasis, on group level […] and that is what we are going to try to interpret and try to improve. And now we are more and more trying to make that step to *individual* patient-level, in which you actually also *discuss* those outcomes with patients (IP 8).

So, according to PROF, the primary purpose of outcome measurements is to foster meaningful improvements. In this line of thought, transparency of outcome information – especially when obligated – is seen as ill-advised. The power of outcome information lies in its learning potential, which threatens to be hampered when its gobbled up by the forces of external accountability.

You first and foremost want to use those numbers well yourself, to see ‘where can we improve and what do I need for that.’ […] If this really wants to work, and if this really wants to play a part in learning and improving, then we should also make those forms of accountability less national, less abstract and far away, but rather much closer by [in the communication] with patients and health insurers (IP 14).

In addition, when faced with the argument that transparency could enable patients to choose between providers, PROF questions whether this would be a positive thing:

That whole idea from a while back, you know the ‘market’ and so on, ‘the patient who chooses’ and that being an important driver of improvements […] although it could help […] that is not the driver. We ourselves have an important responsibility, our own, to make sure that the care is as good as possible […] that should simply remain at the top. And we should not transfer that [responsibility] to a patient, who will then choose with their feet. (IP 14).

To summarize, within PROF, outcome information should primarily be a tool for interprofessional learning and improvement. Patient-choice should not drive improvements, this should come from the internal motivations of professionals. This discourse argues against transparency, which is seen as an external accountability tool, with objectives that are at odds with learning and improvement.

#### Critique (CRI) on outcome information

Within CRI, outcome information can be a useful source – among others – that may fuel the conversations and relations amongst professionals and between and professionals and their patients.

If you would really be willing to use it as an indication, as a start of a conversation, then it could, of course, be beneficial. When that standardized information is not the norm, but rather a tool to start the conversation (IP 22).

A big concern within CRI though, is that outcome data will not nourish conversations but instead stifle medical practice by becoming the oversimplified standard of good care, ‘a reduction of reality’ (IP 16). This will particularly be the case when external parties such as insurers will hold providers accountable for their outcomes:

[E]ach number is valuable, but needs a story. You need more. It’s only part of the story. And my big fear for VBHC and outcome-based payment is reductionistic assessment. So, pretending as if it is simple, based on numbers. And that is bad for health care (IP 16).

Typical for this critical discourse, the rationale relates to the stifling effects of standardization on a personalized approach to care:

As an accountability tool, and with its cost-reducing promises related to the role of insurers, outcome information will very much limit the potential of the patient-doctor conversation. Instead of a helpline, it will grow into a mandatory requirement that must be met, just like the current guidelines and protocols (IP 22).

Within CRI, transparency of outcomes is perceived disturbingly, and the idea that this might lead to better health care is refuted. Instead, CRI presupposes that the *effects* of transparency are undesirable.

When you make all that data transparent to everyone, then certain mechanisms will come into effect, which causes all kinds of manipulations on that data to take place. Therefore, I think, that’s something that you don’t want (IP 25).

Then you’ll brush up those numbers and you’ll get a nice feigned reality (IP 16).

In sum, within CRI, outcome information is regarded as a potentially useful helpline within the patient-doctor conversation and could also serve interprofessional learning. However, standardized measurements will never tell the whole story of the myriad intricacies that correlate in the search for good care. Such numbers should therefore not become the standard by which external parties judge healthcare providers. Accordingly, CRI argues against the transparency of outcomes.

### Common ground

While the statements on measuring and reporting outcomes reveal the starkest differences between the four discourses, common ground can be found. Within each discourse, it is stated that outcome information should eventually be used *within the doctor’s office* and become part of the shared decision-making process regarding medical treatments. Moreover, within each discourse, shared decision-making (SDM) is perceived to be a core element of VBHC. This incorporation of SDM into VBHC is significant since SDM was by no means a defining characteristic of the ‘original’ concept [4, 5, 31, 32]. As will unfold below, this emphasis on SDM goes hand in hand with a redefined understanding of patient value.

#### SDM as a core element of VBHC

A prevailing idea in the Netherlands is that the specific outcomes that matter to patients will often vary greatly between individuals. This is where SDM nicely blends in: it is framed as a mechanism to address the specific needs and interests of individual patients.

[T]o us, it [VBHC] really starts with the right conversation in the doctor’s office, so that is the basis to us. […] And that is let’s say, really the individual care provision […]. And that’s why I think that in the doctor’s office you need to very carefully look at ‘what’s important for this individual patient’ (IP 18).

It is by relating the notion of patient value so strongly to the individual and his or her medical decisions, that SDM is brewed into the idea of VBHC. Although a few of our interviewees stated that there are differences between the two frameworks, the bulk of them portrayed SDM as a key element of VBHC. Several participants regard SDM as the very core of VBHC (IP 2, 14, 15, 17, 18, 21, 25) a viewpoint best illustrated by the statement that ‘shared decision-making is the *condicio sine qua non* of value-based health care’(IP 17). Similarly, multiple official publications – e.g. government reports [26, 29], insurer publications [33], and statements by provider associations [27, 34] – also showed a deep entanglement of the two.

As mentioned, the fusion of VBHC with SDM occurs in all discourses. Within the *Patient Empowerment* discourse (PEMP), SDM is seen as a crucial component of VBHC – as a tool for strengthening the position of patients. The idea here is that a genuinely *shared* decision-making process would be the embodiment of the improved position of patients:

What it is all about is that every patient must be able to participate in decisions about their treatment, on an equal footing. […] Shared decision-making requires a different, equivalent interplay with patients (IP 25).

The *Governance* discourse (GOV), also embraces SDM, but primarily as a way to actively incorporate patients in VBHC – as a mechanism to incentivize providers:

By making the value of care the central focus, and by aiming the incentives in healthcare at it, a common goal emerges for patients, care providers and healthcare insurers [33].

But I think that if we truly want patients to use it, that we should really go all-in on Shared Decision-Making. Namely, the fact that patients will realize that they can choose under those circumstances (IP 9).

In addition, the *Professionalism* discourse (PROF) also perceives SDM as a crucial component of VBHC – as a methodology. Within this discourse though, it primarily becomes a way to improve healthcare delivery by customizing medical treatments:

The patient is our partner in value-based health care. Together with the patient, the healthcare professional discusses what really matters to him or her. Based on personal treatment objectives, healthcare professional and patient together decide on the treatment that will be followed. […] That way, we deliver customized care for each unique patient [34].

Lastly, within *Critique* discourse (CRI), SDM is basically seen as the uniquely positive aspect of VBHC – as a dogma of manufacturability – since ideally, this could enable the personalization of care:

In a kind of positive explanation, I would consider VBHC as an attempt to, what I find nice about it is that it could lead to a much more personalized form of care. That’s what I really consider its most important aspect. […] However, it then concerns, each time, the individual patient. What does that one need? (IP 25).

SDM is thus construed to be a key element of VBHC within each discourse (albeit on slightly different terms). This incorporation not only emphasizes the importance of the patient-doctor relationship for generating value, it also insists on recognizing the patient as an *individual*. Together, this emits a conceptualization of patient value that alters from how the concept was initially put forth.

#### Redefining value

Our study shows a trend in which value is being redefined. The original concept – with the fraction $value=\frac{outcomes}{costs}$ – is regarded as too narrow and too economic.

[W]e considered the approach based solely on Porter too narrow. So, defining value as outcomes versus costs […] we really found that to be too narrow (IP 14).

In the Netherlands, patient value is discursively framed not so much as a strategic goal for the healthcare system [4, 35], but as something that ought to emerge from an interactive patient-doctor relationship which tends to the individual needs of each patient. Within this revised understanding of patient value, the *conversation* between a medical professional and a patient gains significance:

[T]he one-on-one conversation with the patient. That’s where we really want to emphasize ‘what do you find important as a patient?’ You as an individual. [T]here’s a leap from population to individual, and it’s taken in that conversation (IP 14).

‘A leap from population to individual.’ This metaphorical leap, we believe, is illustrative of the manner in which patient value is being reconfigured in the Netherlands; where value in health care is perceived to be not so much determined (economically) by a set of aggregated outcome data that come at a certain price, but rather becomes a matter of informed customization in the doctor’s office.

## Discussion

In the growing body of literature on VBHC, the work and impact of Michael Porter is inescapable. Indeed, it is hard to even find papers that mention VBHC but do not refer to Porter (not impossible though e.g. [36]). However, as should be clear from the introduction of this paper, assuming that this implies a coherent conception of VBHC would be misguided. Instead, VBHC is conceptualized ambiguously in scholarly work [1, 3, 7], which is mirrored in multifarious and fragmented implementation efforts [2, 9, 12] and shines through in our discourse analysis.

Our research indicates that the ambiguity surrounding VBHC is largely due to the concept being *perceived differently through different frames of reference* – which rest upon often deeply rooted presuppositions. VBHC is not, in essence, a particularly ambiguous concept – at least *not necessarily more or less than other concepts*. Instead, the concept is perceived differently by a variety of individuals and organizations, who employ different frames of reference, which manifest themselves in the way they think and talk about value in health care. Therefore, while it is certainly possible that some scholars ‘miss the point’ [1] when writing about VBHC, we argue here that underlying presuppositions *frame one’s point*. In other words, assumptions confine aims, perceptions and (mis)representations [13, 14].

Next to mapping VBHC’s conceptual ambiguity, our discourse analysis aimed to uncover *the way(s) VBHC is interpreted by actors and organizations that monitor and influence the quality of care in the Netherlands.* We identified four discourses, each characterized by a set of statements and particular lines of reasoning; conjoined by underlying presuppositions (see [17, 18]).

In the *Patient Empowerment* discourse (PEMP), VBHC is chiefly portrayed as a strategy for strengthening the position of patients regarding their medical decisions. PEMP is the articulation of VBHC and the foundational presupposition that patients need to be empowered, since healthcare providers may have their own interests. The *Governance* discourse (GOV) also builds on the assumption that the intrinsic motivations of providers are not necessarily aligned with the goal of patient value. Within GOV, however, this issue is seen as best addressed by steering and incentivizing providers. Accordingly, within GOV, VBHC is primarily adopted as a mechanism to incentivize care providers toward better outcomes and lower costs. By contrast, the *Professionalism* discourse (PROF) is a manifestation of the presupposition that the intrinsic motivations of medical professionals are already in line with those of patients and the notion of patient value. In PROF, VBHC is construed as a methodology for healthcare delivery, emphasizing continuous improvement. Lastly, in the *Critique* discourse (CRI), VBHC is deemed to be a dogma of manufacturability; one that mistakenly claims that the highest quality care can be produced and measured systematically. CRI presupposes that health care forms a sphere of intricacies that cannot be adequately addressed by an approach that departs from a standardized notion of value.

In the Netherlands, despite the discursive divergencies, there are two general ways in which each discourse contrasts with the pioneering literature on VBHC. Firstly, shared decision-making (SDM) is deeply ingrained in the conception of VBHC. While it would be hard to argue that SDM forms a central element of VBHC as it was originally outlined [cf. 4, 5, 31, 32], it could very well be argued that SDM constitutes a major component of VBHC *in the Netherlands*. Recently, other scholars have advocated the incorporation of SDM and individual patient preferences when implementing and evaluating VBHC [37], which may reflect an earlier call to combine biomedical individualization with the relational aspects of SDM, in order to truly personalize medicine [38].

Secondly, the issue of *competition* among providers has been conspicuously absent in most of the texts we analyzed. This absence is noteworthy, since the idea of value-based competition forms the undeniable cornerstone of Porter and Teisberg’s thesis [4, 31, 39]. In fact, in *Redefining Health Care* (2006) [4], the term ‘value-based health care’ appears exactly once (as an adjective, p. 162). Instead, throughout the book, the authors speak of value-based competition. Interestingly, while scholars often refer to Porter and Teisberg’s (2006) work as the pioneering text on VBHC, the preeminence of *competition* is rarely acknowledged sufficiently (a notable exception being Groenewoud et al. 2019 [7]). This confirms our earlier claim that – in academic texts as well – frameworks of reference tentatively determine what VBHC is perceived to be.

Furthermore, the label ‘value-based health care’ will not only be perceived and utilized diversely in thought and (academic) writing, it will also continue to engender varying and probably contrasting practical initiatives, which will be evaluated differently, according to different standards [40–42]. Therefore, although we tend to agree with the notion that VBHC requires empirical evidence [9, 40, 42], our paper conveys a primal issue; namely: *evidence of what?* In light of differing perceptual frameworks and diverging discourses, we strongly urge scholars to be particularly deliberate when researching and writing in relation to VBHC.

Our study has at least two important limitations. Firstly, our analysis focused specifically on the Netherlands: its particular healthcare system and health policy climate may clearly inhibit the comparability of our results. Moreover, scholars have stated that the way in which VBHC (as a concept) manifests itself is contingent upon specific local and regional complexities [3, 43]. As our study shows that the concept of VBHC is being perceived differently *within* the Dutch context, we strongly encourage future research regarding discourses on VBHC within other systems and regions.

The second main limitation of this study concerns the fact that we have focused primarily on what is said and written, not on what is acted out. It goes without saying that people’s actions may contradict their words; it was, however, beyond the scope of this study to investigate how and to what extent the four discourses relate to actual decisions and practices concerning VBHC. We therefore advocate additional research regarding practical implementations of VBHC.

## Conclusion

Our current study is vital since our discourse analysis demonstrates that the meaning of VBHC – *including its perceived implications for action* – depends greatly on the frame of reference a certain actor or organization brings to bear in relating to this concept. Moreover, the various discourses (which may differ between countries) will shape current and future debates on VBHC [44, 45]. The decisions that result from these debates may have a significant impact: they will regulate what exactly will be measured and reported when it comes to outcomes; these decisions will thus affect how providers will be held accountable; they will construct standards of good (i.e. valuable) care; they may influence the way insurers will structure their payment; they could influence patients’ decisions, both regarding treatments and regarding providers; plus they may have an impact on the conversations in the doctor’s office.

Accordingly, these discourses will profoundly shape the diverse manners in which VBHC moves from an abstract concept to the practical provision and administration of health care. Therefore, policymakers, healthcare administrators and practitioners would be wise to at least take the validity of different logics and their entrenched presuppositions into consideration when they truly aim for more value for patients. We hope our study may contribute to this end.

## Supplementary information

**Additional file 1.** A COREQ checklist.

**Additional file 2.** Interview guide.

## Data Availability

No additional data are available.

## Abbreviations

VBHC: Value-based health care
SDM: Shared decision-making
PEMP: Patient Empowerment discourse;
GOV: Governance discourse
PROF: Professionalism discourse
CRI: Critique discourse.

## References

[1] Fredriksson JJ, Ebbevi D, Savage C (2015). Pseudo-understanding: an analysis of the dilution of value in healthcare. BMJ Qual Saf.

[2] Colldén C, Hellström A (2018). Value-based healthcare translated: a complementary view of translation. BMC Health Serv Res.

[3] Bonde M, Bossen C, Danholt P (2018). Translating value-based health care: an experiment into healthcare governance and dialogical accountability. Sociol Health Illn.

[4] Porter ME, Teisberg EO (2006). Redefining health care: creating value-based competition on results.

[5] Porter ME (2010). What is value in health care. N Engl J Med.

[6] Nilsson K, Bååthe F, Andersson A, Wikström E, Sandoff M (2017). Experiences from implementing value-based healthcare at a Swedish University hospital – an longitudinal interview study. BMC Health Serv Res.

[7] Groenewoud AS, Westert GP, Kremer JAM (2019). Value based competition in health care’s ethical drawbacks and the need for a values-driven approach. BMC Health Serv Res.

[8] Busink E, Canaud B, Schröder-Bäck P, Paulus A, Evers S, Apel C, Bowry S (2019). 2019. Chronic kidney disease: exploring value-based healthcare as a potential viable solution. Blood Purif.

[9] Ebbevi D (2017). Value-based health care: challenges in moving forward [Ph.D.].

[10] Liu T, Bozic K, Teisberg E (2016). Value-based healthcare: person-centered measurement: focusing on the three c’s. Clin Orthop Relat Res.

[11] Conrad DA (2015). The theory of value-based payment incentives and their application to health care. Health Serv Res.

[12] Andersson A, Bååthe F, Wikström E, Nilsson K (2015). Understanding value-based healthcare – an interview study with project team members at a Swedish university hospital. J Hosp Adm.

[13] Peterson J (1999). Maps of meaning: the architecture of belief.

[14] Peterson J, Flanders J (2002). Complexity management theory: motivation for ideological rigidity and social conflict. Cortex..

[15] Geertz C (1973). The interpretation of cultures: selected essays.

[16] Boivin A, Green J, van der Meulen J, Légaré F, Nolte E (2009). Why consider patients’ preferences?: a discourse analysis of clinical practice guideline developers. Med Care.

[17] Cheek J (2004). At the margins?: discourse analysis and qualitative research. Qual Health Res.

[18] Watson T (1994). In search of management: culture, chaos and control in managerial work.

[19] Alvesson M, Kärreman D (2000). Varieties of discourse: on the study of organizations through discourse analysis. Hum Relat.

[20] Hodges B, Kuper A, Reeves S (2008). Qualitative research: discourse analysis. BMJ..

[21] Newton T (1998). Theorizing subjectivity in organizations: the failure of Foucauldian studies?. Organ Stud.

[22] Greenhalgh T, Procter R, Wherton J, Sugarhood P, Shaw S (2012). The organising vision for telehealth and telecare: discourse analysis. BMJ Open.

[23] Lang A (2019). The good death and the institutionalisation of dying: an interpretive analysis of the Austrian discourse. Soc Sci Med.

[24] Green J, Thorogood N (2009). Qualitative methods for health research.

[25] Schippers E. Kamerbrief over Samen beslissen [Internet]. Rijksoverheid.nl. 2015. Available from: https://www.rijksoverheid.nl/documenten/kamerstukken/2015/10/29/kamerbrief-over-samen-beslissen. [cited 10 March 2020].

[26] Ministry of Health Welfare and Sports. Ontwikkeling uitkomstgerichte zorg 2018–2022 [Internet]. Rijksoverheid.nl. 2018 [cited 10 March 2020]. Available from: https://www.rijksoverheid.nl/documenten/rapporten/2018/07/02/ontwikkeling-uitkomstgerichte-zorg-2018-2022.

[27] Federation of Medical Specialists. Standpunt Value Based Healthcare [Internet]. Federatie Medisch Specialisten. 2018 [cited 10 March 2020]. Available from: https://www.demedischspecialist.nl/nieuws/standpunt-value-based-healthcare.

[28] Federation of Medical Specialists. Vision document: Medical Specialist 2025 [Internet]. Demedischspecialist.nl. 2017 [cited 10 March 2020]. Available from: https://www.demedischspecialist.nl/sites/default/files/FMS_visiedoc_MS2025%28eng%29_2017_PL_v02%28lr%29.pdf.

[29] National Health Care Institute. Rapport 'Meer patiëntregie door meer uitkomstinformatie in 2022′ [Internet]. Zorginstituutnederland.nl. 2018 [cited 10 March 2020]. Available from: https://www.zorginstituutnederland.nl/publicaties/rapport/2018/06/28/rapport-meer-patientregie-door-meer-uitkomstinformatie-in-2022.

[30] Menzis. Visie 2020: Waardegerichte Zorginkoop [Internet]. Menzis voor zorgaanbieders. 2019 [cited 10 March 2020]. Available from: https://www.menzis.nl/zorgaanbieders/zorginkoop).

[31] Porter ME, Teisberg EO (2007). How physicians can change the future of health care. Jama..

[32] Porter ME (2008). Value-based health care delivery. Ann Surg.

[33] Menzis. Waardegerichte zorginkoop borstkanker [Internet]. Menzis.nl. 2018 [cited 21 October 2019]. Available from: https://www.menzis.nl/zorgaanbieders/-/m/publieke-sites/menzis/zorgaanbieders/downloads/zorgsoorten/medisch-specialistische-zorg/contractering/inkoopbeleid-2019/prospectus-waardegericht-inkopen-borstkankerzorg_12112018.pdf.

[34] Netherlands Federation of University Medical Centers. Position Paper Waardegedreven Zorg [Internet]. Nfukwaliteit.nl. 2019 [cited 4 June 2019]. Available from: https://nfukwaliteit.nl/pdf/NFU-Position_Paper_Waardegedreven_Zorg.pdf.

[35] Porter M, Lee TH (2013). The strategy that will fix health care. Harv Bus Rev.

[36] Moriates C, Valencia V, Stamets S, Joo J, MacClements J, Wilkerson L (2019). Using interactive learning modules to teach value-based health care to health professions trainees across the United States. Acad Med.

[37] Van Deen W, Nguyen D, Duran N, Kane E, van Oijen M, Hommes D (2016). Value redefined for inflammatory bowel disease patients: a choice-based conjoint analysis of patients’ preferences. Qual Life Res.

[38] Burke W, Brown Trinidad S, Press N (2014). Essential elements of personalized medicine. Urol Oncol.

[39] Porter ME, Teisberg EO (2004). Redefining competition in health care. Harv Bus Rev.

[40] Garvelink M, Van der Nat P (2019). Moving forward with value based healthcare: the need for a scientific approach. Eur J Surg Oncol.

[41] Van Egdom L, Lagendijk M, Van der Kemp M, Van Dam J, Mureau M, Hazelzet J (2019). Implementation of value based breast cancer care. Eur J Surg Oncol.

[42] Van Egdom L, Hazelzet J, Koppert L (2019). Reply to: Moving forward with value-based healthcare: the need for a scientific approach. Eur J Surg Oncol.

[43] Dainty K, Golden B, Hannam R, Webster F, Browne G, Mittmann N (2018). A realist evaluation of value-based care delivery in home care: the influence of actors, autonomy and accountability. Soc Sci Med.

[44] Schmidt V (2008). Discursive institutionalism: the explanatory power of ideas and discourse. Annu Rev Polit Sci.

[45] Stevens M, Wehrens R, De Bont A (2018). Conceptualizations of big data and their epistemological claims in healthcare: a discourse analysis. Big Data Soc.

