# Supplementary material for: Redefining value: a discourse analysis on value-based health care
Source: BMC Health Serv Res. 2020 Sep 14;20:862. doi: 10.1186/s12913-020-05614-7 (PMC7488985; doi:10.1186/s12913-020-05614-7)
Supplement: Supplementary file 1 — Additional file 1. A COREQ checklist. [file 12913_2020_5614_MOESM1_ESM.pdf]

Supplementary file 1. Consolidated criteria for reporting qualitative studies (COREQ): 32-item checklist

Topic and Item No.	Guide Questions/Description	Reported on Page No.
**Domain 1: research team and reflexivity**		
Personal Characteristics		
1. Interviewer/facilitator	Which author/s conducted the interview or focus group?	Gijs Steinmann (Reported on page 41)
2. Credentials	What were the researcher's credentials? E.g. PhD, MD	Gijs Steinmann, MA, Msc; Hester van de Bovenkamp, PhD; Antoinette de Bont, PhD; Diana Delnoij, PhD
3. Occupation	What was their occupation at the time of the study?	GS PhD Candidate; HB Associate Professor; DD Professor; AB Professor
4. Gender	Was the researcher male or female?	GS male; HB, AB & DD female
5. Experience and training	What experience or training did the researcher have?	GS: university degrees in cultural anthropology, with in-depth interviews conducted for bachelor and master theses.
Relationship with participants		
6. Relationship established	Was a relationship established prior to study commencement?	No
7. Participant knowledge of the interviewer	What did the participants know about the researcher? *e.g. personal goals, reasons for doing the research*	Participants were informed about the projects' objectives (including the first author's PhD-trajectory) in an invitational e-mail and again prior to the interview.
8. Interviewer characteristics	What characteristics were reported about the interviewer/facilitator? e.g. *Bias, assumptions, reasons and interests in the research topic*	It was disclosed that the project was established through the collaboration of Zorginstituut (funder) and the Erasmus School of Health Policy & Management.
**Domain 2: study design**		
Theoretical framework		
9. Methodological orientation and Theory	What methodological orientation was stated to underpin the study? *e.g. grounded theory, discourse analysis, ethnography, phenomenology, content analysis*	Discourse analysis (Methods p. 7-10)
Participant selection		
10. Sampling	How were participants selected? *e.g. purposive, convenience, consecutive, snowball*	Purposive (p. 9)
11. Method of approach	How were participants approached? *e.g. face-to-face, telephone, mail, email*	E-mail
12. Sample size	How many participants were in the study?	23 (p. 9)
13. Non-participation	How many people refused to participate or dropped out? Reasons?	1. "Too busy"

Setting		
14. Setting of data collection	Where was the data collected? *e.g. home, clinic, workplace*	Workplace of participants
15. Presence of non-participants	Was anyone else present besides the participants and researchers?	No
16. Description of sample	What are the important characteristics of the sample? *e.g. demographic data, date*	NA
Data collection		
17. Interview guide	Were questions, prompts, guides provided by the authors? Was it pilot tested?	Yes (interview guide added separately to manuscript submission).<br><br>One pilot interview with a colleague was conducted to test the questions.
18. Repeat interviews	Were repeat interviews carried out? If yes, how many?	No
19. Audio/visual recording	Did the research use audio or visual recording to collect the data?	Audio recorded (p. 9)
20. Field notes	Were field notes made during and/or after the interview or focus group?	No
21. Duration	What was the duration of the interviews or focus group?	Average duration 55 minutes (p. 9)
22. Data saturation	Was data saturation discussed?	Yes (p. 9)
23. Transcripts returned	Were transcripts returned to participants for comment and/or correction?	No
**Domain 3: analysis and findings**		
Data analysis		1 actual coder, transcripts read by 2,
24. Number of data coders	How many data coders coded the data?	coding process discussed by 4 (p. 10)
25. Description of the coding tree	Did authors provide a description of the coding tree?	Yes (p. 10)
26. Derivation of themes	Were themes identified in advance or derived from the data?	Derived from data (p. 10)
27. Software	What software, if applicable, was used to manage the data?	Atlas.ti (p. 10)
28. Participant checking	Did participants provide feedback on the findings?	Yes (p. 41)
Reporting		
29. Quotations presented	Were participant quotations presented to illustrate the themes / findings? Was each quotation identified? *e.g. participant number*	Yes (Results p. 11-29)
30. Data and findings consistent	Was there consistency between the data presented and the findings?	Yes (Results p. 11-29)
31. Clarity of major themes	Were major themes clearly presented in the findings?	Yes (Results p. 11-29)
32. Clarity of minor themes	Is there a description of diverse cases or discussion of minor themes?	Yes (Results p. 11-29)
