# Supplementary material for: Redefining value: a discourse analysis on value-based health care
Source: BMC Health Serv Res. 2020 Sep 14;20:862. doi: 10.1186/s12913-020-05614-7 (PMC7488985; doi:10.1186/s12913-020-05614-7)
Supplement: Supplementary file 2 — Additional file 2. Interview guide. [file 12913_2020_5614_MOESM2_ESM.pdf]

Supplementary file 2. Interview guide

Introduction:

Research background & objectives. Permission to record. Informed consent.

Opening question:

- What do you understand by 'value-based health care'?

Main questions:

- What do you understand by 'value' ?
- Should we measure outcomes? Why/Purpose? What info? For whom?
- How is/should this info eventually be used? By whom?
- Should we measure costs? Why/Purpose? How/what info?
- How do you view the relation between VBHC and cost-efficiency?
- How does shared decision-making (SDM) relate to VBHC?
- Should we implement VBHC in NL? Why (not)?

Topic list

- Patient Value
- Outcomes & Costs (measurement & reporting)
- Shared decision-making (SDM)
- IPU - Integrated Practice Units (full cycle – medical condition)
- Bundled Payments
- Purchasing (value-based / role insurer)
- Choice
- Competition (value-based)

Optional/extra:

- Should we change the way we organize/structure our healthcare delivery (specialism, silos)?
- Should we change the wat we pay for healthcare delivery (reward value vs volume)?
- Do you believe in competition among healthcare providers?
- Should we make outcome measurements transparent?
- Do you see VBHC having a significant on future health care in the Netherlands (explain)?
- What is your perspective on the quality of care in the Netherlands? On what basis do come to this viewpoint?
- Do you believe there are significant differences between providers (quality)? How to best address this?
